# Foam coarsening under a steady shear: interplay between bubble rearrangement and film thinning dynamics†

**DOI:** 10.1039/d2sm01618d

**Published:** 2023-02-17

**Authors:** Arnaud Saint-Jalmes, Corentin Trégouët

**Affiliations:** a Univ Rennes, CNRS, IPR (Institut de Physique de Rennes), UMR 6251 F-35000 Rennes France arnaud.saint-jalmes@univ-rennes1.fr

## Abstract

Aqueous foams are unstable and age by drainage and coarsening. Today, these effects are well described, as also their impact on foam properties. In that respect, the foam viscoelastic properties evolve in time as a consequence of coarsening which tends to increase the mean bubble size. Here, we investigate the reverse coupling, and study if and how the continuous flow of a foam can impact its dynamics of coarsening. We introduce a new protocol where brief oscillatory measurements are inserted during a constant steady shear, allowing us to monitor the relative variation of the bubble size with time (obtained from the one of the elastic modulus *G*′) as a function of the applied shear rate. It turns out that the coarsening rate is strongly impacted by the applied shear: this rate is continuously reduced above a critical shear rate, which itself decreases with the bubble size. This coarsening-rate reduction is interpreted as the result of out-of-equilibrium and shear-dependent film thicknesses, being higher than at rest. The critical shear rate, above which films are dynamically sustained at higher thickness than at equilibrium, emerges from the competition between the rate of rearrangements and the time required to drain the thick film created during the rearrangement. We thus report here a first experimental proof and measurements of out-of-equilibrium film thicknesses within a sheared foam, and of the impact this has on coarsening.

## Abstract 2

Above a given threshold, foam coarsening is reduced by an applied shear.

## Graphical abstract

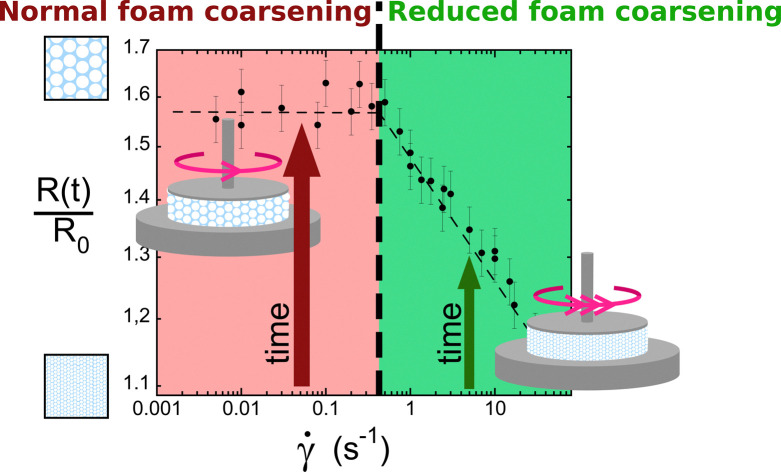

## 1 Introduction

Aqueous foams are dispersions of gas bubbles within a continuous liquid matrix.^1,2^ The resulting dispersion has stunning properties when compared to the separated initial gas and liquid phases, in terms of mechanics,^3,4^ acoustics^5^ or optics.^6^ As a consequence, such specific features make them the most efficient material in many applications. Additionally, on an everyday basis, they are encountered in food and cosmetics, mostly because of their viscoelastic and yielding properties.

Previous studies show that the rheological properties of aqueous foams depend on two crucial parameters: the bubble size and the liquid fraction, describing to what extent the bubbles are packed.^3,4,7–11^ This packing results in polyhedral shapes of bubbles, and the creation of thin liquid films separating them.^12,13^ At low enough liquid fractions, once bubbles are packed, a foam develops a viscoelastic behavior, with a dominant elastic modulus *G*′ below a yield strain, and above which it starts to flow. The intrinsic scale for this elasticity is the Laplace pressure, *P* = *σ*/*R* (with *σ* the surface tension and *R* a typical bubble radius), and *G*′ is then simply set by *P*, pondered by the liquid fraction.^3,7,8,10^

To better understand the foam viscoelasticity and its flow properties, the links between the macroscopic rheological properties and the dynamics at the scale of the bubble have also been studied, evidencing the central role of topological bubble rearrangements, called T1.^14–20^

Despite these recent advances, many issues are still pending on foam rheology, for instance on wall slipping^21,22^ or on flow uniformity (like shear banding^23^). Also, the local origins of dissipation – which set the viscous modulus *G*′′ and the foam viscosity – and the links to the chemical formulation (*via* the interfacial and bulk viscoelasticity) still need to be fully understood.^21,24–29^

Complexity also arises in foams due to their aging, as foams are metastable materials and irreversibly evolve by drainage,^30,31^ coarsening due to gas diffusion,^32–39^ and film rupturing.^40,41^ The coarsening in aqueous foams shares many features with the more generic ripening phenomenon,^42–45^ which occurs for a wide range of systems, resulting in a characteristic lengthscale which grows with time (to minimize interfacial energy). Specifically, for aqueous foams, remaining questions mostly deal with how the dynamics depends on the liquid fraction,^39,46,47^ and on how coarsening can be tuned by the chemical formulation.^31,48^

As for many other soft glassy materials (like emulsions or colloidal gels), aging and rheology are strongly coupled;^26,49–53^ for foams, the elastic modulus *G*′ (Pa) evolves in time because it depends on the liquid fraction and on the bubble size, which are respectively decreasing (drainage) and increasing with aging (coarsening). As a consequence, for a dry foam aging mostly by coarsening, monitoring *G*′ over time has been used to indirectly recover the standard scaling behavior 
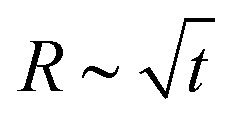
,^49–51,53^ evidenced by other techniques.^33,54^ Comparatively, much less is understood about the time evolution of *G*′′ (Pa) and of the foam viscosity,^11,26,55^ and on which parameters set their aging.

Since aging impacts rheology, one can then wonder if this coupling can operate in the other direction: more precisely, if a continuous flow can modify the aging of foams. Here we study the coarsening of foams under flow by measuring the evolution in time of the elastic modulus, *G*′ as a continuous steady shear is also imposed. We show that beyond a threshold shear rate, coarsening is strikingly slowed down, and that is due to out-of-equilibrium film thicknesses, arising from the local reorganization events triggered by the shear.

## 2 Materials and methods

The experiments consist in shearing a shaving foam (*Gillette*) inside a plate–plate configuration using an Anton Paar MCR-301 rheometer. The diameter of the plate is 75 mm. This foam is known for its reproducible properties in terms of initial bubble radius (*R*≃ 30 mm) and liquid fraction (*ϕ*_l_≃ 7%). Thanks to the small height of the foam sample (2 mm), gravitational drainage is prevented, and aging is only due to coarsening.

**Fig. 1:**
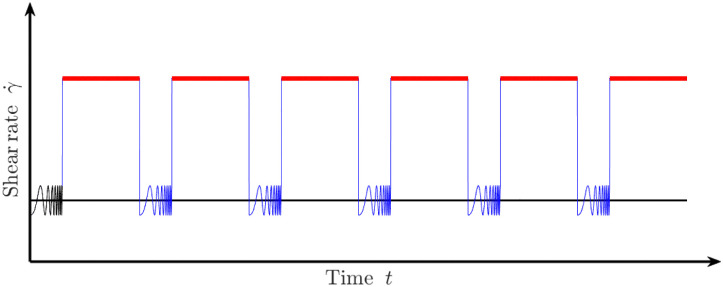
Measurement protocol: the foam is alternatively probed through a frequency sweep (in blue), and continuously sheared at a constant shear rate (in red). The reproducibility of the initial conditions is checked by an initial frequency sweep (in black).

The experimental protocol is illustrated on Fig. 1. For a given sample, we start by performing a first oscillatory frequency-sweep test, with a frequency *f* varied from 0.5 to 5 Hz and at a constant amplitude of 1%. This allows us to measure the initial elastic and viscous moduli, and to check the reproducibility of the samples. Then, a steady shear at a constant rate *γ̇* (in s^−1^) is applied for 120 minutes. But, it is paused every 10 minutes to perform a measurement of the elastic and viscous moduli by the same frequency-sweep test (*f* = [0.5,5]Hz – amplitude of 1%) as the initial one. Each oscillatory measurement takes 4 minutes, and such a measurement is inserted 8 times during the overall experimental run. This same protocol is then performed for a new sample, but with a different shear rate *γ̇*; and these shear rates are finally varied from 0 to 50 s^−1^. Note that the oscillatory measurements are performed over short periods of time, and at very low shear amplitude of strain (well below the yield strain), so that they are not impacting the foam coarsening.

## 3 Results

### 3.1 Elastic modulus

**Fig. 2:**
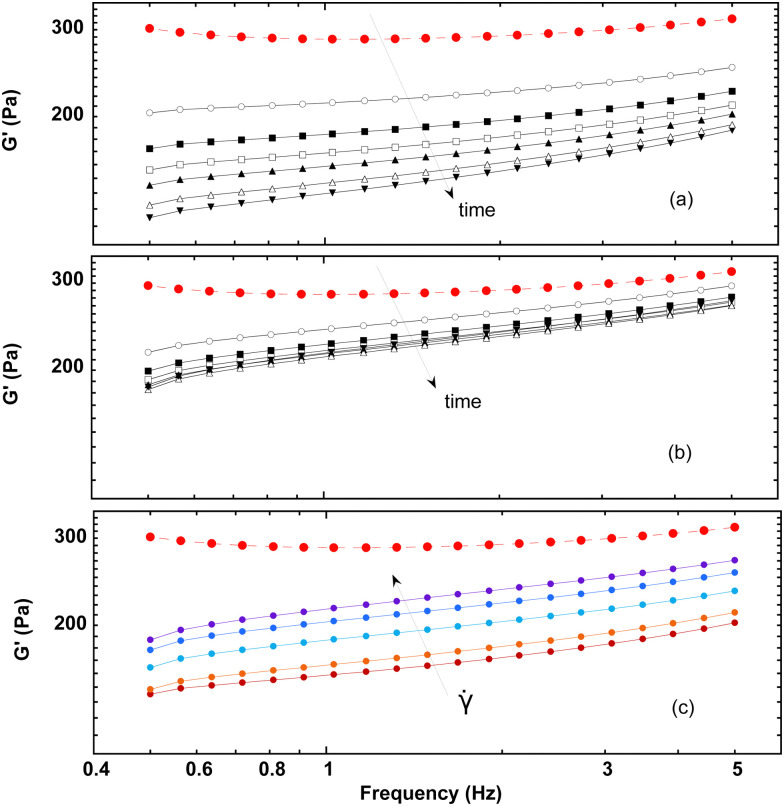
(a) Evolution of the storage modulus with frequency for different aging times (*t* = 200, 1100, 2000, 2900, 3800, 5600, and 7400 s) under a low shear rate (*γ̇* = 0.005 s^−1^); and (b) under a high shear rate (γ̇ = 10 s^−1^). (c) Comparing experiments at the same aging time (here *t* = 3800 s), for different shear rates (*γ̇* = 0.005, 0.35, 1.7, 2.5, and 10 s^−1^). The red dashed line shows the initial measurement for comparison. In this initial measurement, results are reproducible within a maximum of 5% of variation.

Measurements of the elastic modulus *G*′, by successive frequency sweeps are shown in Fig. 2 for shear rates of 0.005 s^−1^ (a) and 10 s^−1^ (b). As already reported, *G*′ is almost constant with the frequency, but decreases with aging. The same qualitative behavior is recovered at a higher shear rate (Fig. 2(b)), but with much less variation in *G*′ over the same timescale. We have found that *G*′ decreases less and less, when the applied shear rate increases. Fig. 2(c) shows another way of presenting these results: we plot our data for different the shear rates, at a fixed aging time. This clearly evidences that the time-dependence of the foam elasticity depends on the applied shear rate.

**Fig. 3:**
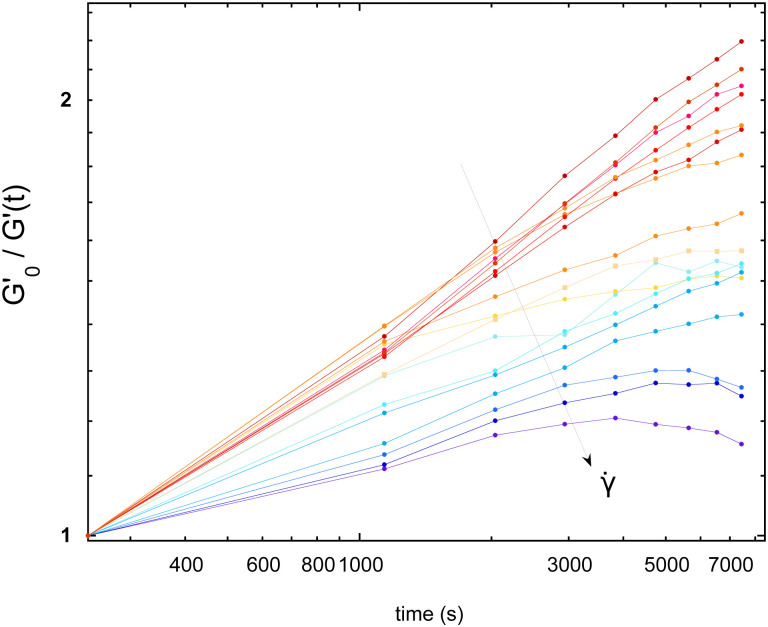
Time evolution (in log–log scale) of the ratio 
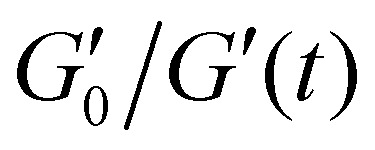
 – at *f*_0_ = 1 Hz – for increasing shear rates (*γ̇* ∈ [0.005, 50] s^−1^) (arrow). 
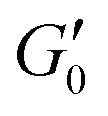
 is the initial elastic shear modulus *G*′(*t* = 0). High shear rates progressively decrease the coarsening dynamics.

In Fig. 3, we selected the frequency *f*_0_ = 1 Hz, and plot *G*′(0)/*G*′(*t*) at *f*_0_, for different shear rates. For the sake of clarity, only a fraction of the measured shear rates are represented in Fig. 3. As stated before, the foam elasticity is set by the Laplace pressure *P*.^10^ More precisely,1
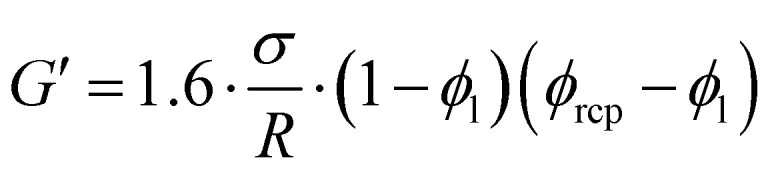
where *ϕ*_l_ is the liquid fraction, and *ϕ*_rcp_ = 0.36 (random close packing). Therefore, a decrease of *G*′ is a direct signature of the increase of *R* over time, and *G*′(0)/*G*′(*t*) = *R*(*t*)/*R*(0). Thus, by measuring the relative variation of *G*′, we get relative variations of *R*. For clarity, *G*′(0) is noted 
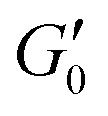
. Fig. 3 shows that the applied shear modifies the bubble growth rate which is set by the coarsening: the higher the shear rate the slower the coarsening.

This effect of a steady shear on the bubble radius has been independently corroborated by bubble size measurements under a microscope, as shown in the ESI.† Comparatively, it is worth noting that inferring the evolution of the bubble radius by rheological measurement is both non-destructive and more accurate, as it is averaged over the whole sample, and thus over thousands of bubbles.

### 3.2 Viscous modulus

**Fig. 4:**
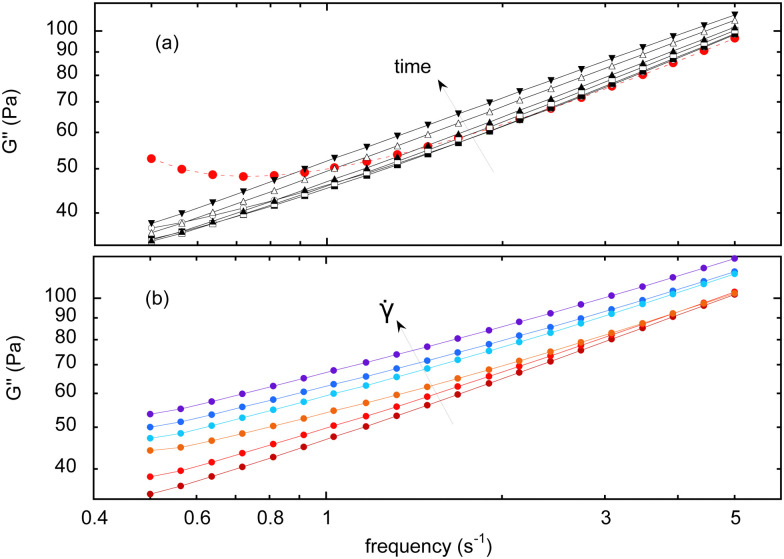
(a) Viscous modulus, *G*′′ obtained by a frequency sweep, at different aging times (*t* = 200, 1100, 2000, 2900, 3800, 5600, and 7400 s), inserted during a shear rate of 0.005 s^−1^. The red dashed curve is the first measurement before any continuous shear is applied. (b) At different shear rates (*γ̇* = 0.005, 0.35, 1.7, 2.5, 10, 30 and s^−1^), for a given time (*t* = 380 s).

The evolution of the viscous modulus *G*′′ with the frequency *f* is given in Fig. 4(a), measured at different aging times and under a shear rate of 0.005 s^−1^. Note that for some given experimental conditions, the variation between various measurements is about 5% of the measured values.

It turns out that the first steady-shear interval strongly alters the shape of the curve, especially at the lowest frequencies. As a consequence, all the measurements – performed after a steady-shear interval – eventually show a scaling behavior, *G*′′ ∼ *f*^*β*^. For the lowest shear rates, the prefactor increases by about 25% during the experimental run, while *β* remains almost constant at a value close to 0.5, consistent with the initial measurement at high frequencies (Fig. 4(a)), and as already reported in the literature.^3^

In parallel, the Fig. 2(b) shows that *G*′′ increases – at a same aging time – for increasing shear rates, meaning that the higher the imposed shear rate, the stronger the variation of *G*′′ with time (which is the opposite of what is found for *G*′). Still, a scaling behavior is always found, but with an exponent *β* varying from 0.5 to 0.38 with the shear rate. More precisely, this exponent is initially almost constant with the shear rate, and mostly decreases for the highest values of shear rates.

### 3.3 Viscosity

**Fig. 5:**
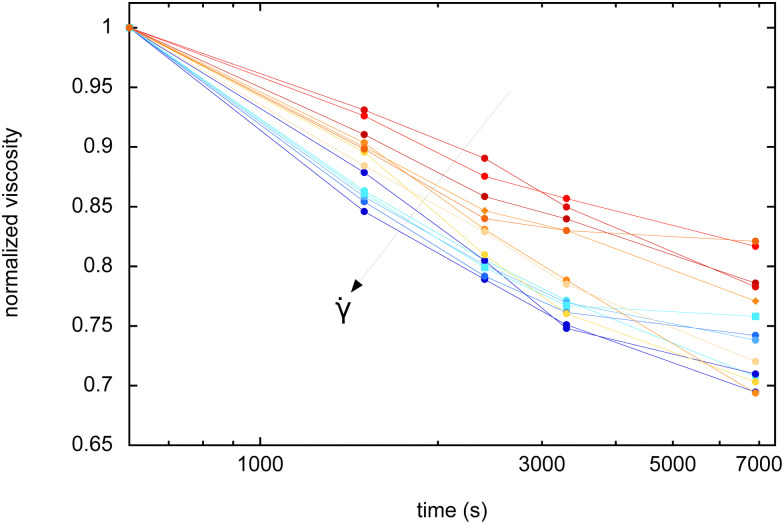
Normalized viscosity (dimensionless) as a function of aging time, for different applied shear rates (*γ̇* ∈ [0.005, 10] s^−1^).

It is also possible to extract complementary information from the steady shear intervals. For all the shear rates, we collect the viscosity *η* (in Pa s), measured in the middle of the intervals (after 5 minutes of shear), and we normalized it by the initial viscosity measured during the first interval. Dimensionless normalized viscosities are reported in Fig. 5 for increasing shear rates: it is found that *η* always decreases with time, but the rate of decrease depends on the shear rate: the higher the imposed shear rate, the higher the rate of decrease of the viscosity.

## 4 Discussion

### 4.1 From the evolution of *G*′: how shear reduces coarsening by adjusting film-thickness

**Fig. 6:**
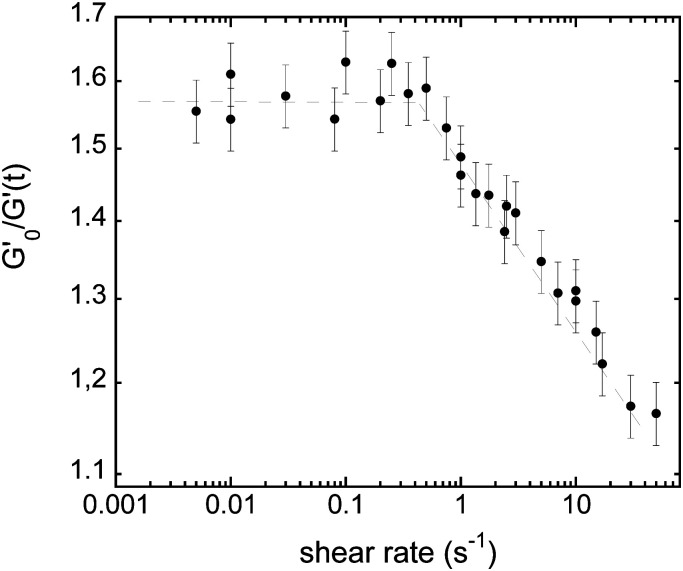
Ratio 
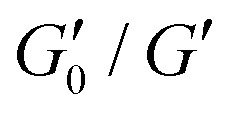
, at a given aging time *t* = 2900 s, as a function of the shear rate. Two regimes can be easily identified: a first regime at small *γ̇* where coarsening is independent of the shear rate (nominal unperturbed coarsening), and a second regime where the coarsening is reduced by the increasing shear rate (reduced coarsening). The transition between the two regimes (crossover of the two asymptotes) defines the critical shear rate *γ̇*_c_.

Compiling data – at any given aging time – leads to the behavior illustrated in Fig. 6. For low shear rates, the dynamics is independent of the shear and the radius reached after a given time does not depend on the applied shear; while above a threshold shear rate, the coarsening is slowed down and the bubble radius decreases with the shear rate.

This allows making an estimation – at each time point – of a critical shear rate *γ̇*_c_, typically ranging from 0.1 s^−1^ to 1 s^−1^.

**Fig. 7:**
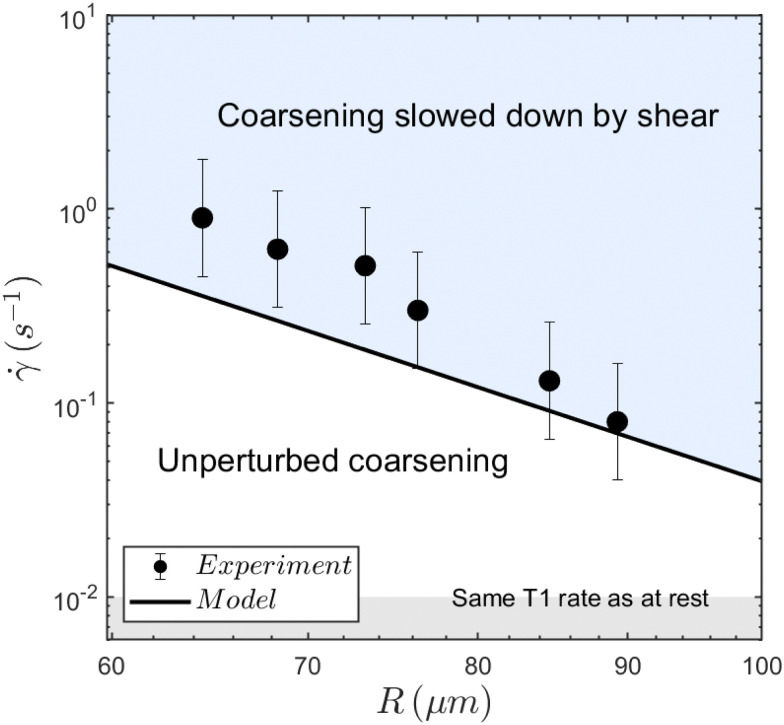
Measured critical shear rates as a function of the bubble radius at the regime transition for the different times, delimiting two regions. The larger the bubble radius, the smaller the critical shear rate. The bubble radius increasing with time, at any given shear rate, the foam always eventually reaches the threshold and enters the slow-coarsening regime. The solid line results from the model yielding eqn (7). The mean absolute deviation of the model is 0.24 s^−1^, and the mean relative deviation is 45%.

As each aging time is associated with a bubble radius, corresponding to the plateau for *γ̇* < *γ̇*_c_ and taking *R*_0_ = 30 mm for the initial bubble size, we can associate the critical shear rate with the corresponding plateau bubble radius *R*(*t*) and plot them in Fig. 7. This provides us with a phase diagram separating regimes where coarsening is nominal (low bubble radius and low shear rate), or reduced (high bubble radius and high shear rate).

First, we recall what sets the coarsening timescale, *t*_c_: *t*_c_ = *R*_0_^2^/(2*D*_eff_*f*(*ϕ*_l_)), with *R*_0_ the bubble radius at *t* = 0. The function, *f*, describes the surface fraction of thin films covering a bubble; it falls down to 0 when bubbles are no longer packed (*ϕ*_l_ > 0.36) and tends to 1 for extremely dry foams (*ϕ*_l_ ≪ 1).^1,2,12^ The effective diffusion coefficient *D*_eff_ (in m^2^ s^−1^) depends on the properties of the chemicals as discussed in^32^2
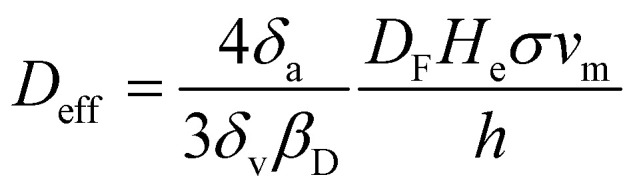
Here, *σ* is the surface tension (mN m^−1^), *h* is the thin film thickness (set by the drainage due to the liquid-menisci capillary suction), and *δ*_a_, *δ*_v_ and *β*_D_ are dimensionless geometrical constants. Linked to the gas, *v*_m_ is the ideal gas molar volume (m^3^ mol^−1^), *D*_F_ is the diffusivity of the gas inside the thin liquid films (in m^2^ s^−1^), and He is the gas Henry constant (in mol (m^−3^ Pa^−1^)), reflecting the solubility of the gas.

Looking at this previous equation, most of the parameters are not expected to change when the foam is sheared. Gas diffusivity can be affected by the surfactant nature and concentration,^32^ but it fails to explain the observed phenomenon for two reasons: 1 – it implies that surfactant adsorption would slow down air diffusion. As a consequence, Ostwald ripening would be faster for young interfaces (with few surfactants) than for old saturated interfaces. Consequently, interface renewal during shear should accelerate ripening, which is the opposite of the observed effect. 2 – the foaming solution is very concentrated in surfactants, and the adsorption time is therefore probably very short: at the time scale of the film life-time, we believe that the monolayer is always almost at equilibrium, so the effect of monolayer concentration modification should be of very small amplitude.

The film thickness, on the other hand, can be shear-dependent. Assuming that it is the only parameter varying significantly in eqn (2), the relative thickness *h* can be extracted from the initial growth rate of 
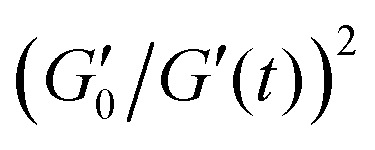
 with the data of Fig. 3. The thickness extracted from the coarsening early kinetics shows an increase by a factor 3 at high shear rate compared to the low shear-rate thickness (see the ESI†).

In fact, shearing a foam is known to induce topological rearrangements called T1. Such a rearrangement consists of a change in the bubble neighbors, and hence in the disappearance of films and the creation of new ones. The newly created film is initially thicker (at *h*_max_) than a film at equilibrium that had time to be drained by the capillary suction of its surrounding menisci to *h*_min_.

If there is no T1, drainage makes the thickness converge to a minimum equilibrium thickness *h*_min_, while just after a T1, the film reaches its maximum thickness *h*_max_.

To determine whether the topological rearrangement may have an impact on the coarsening rate *via* the changes of the film thickness, one has to compare the time *τ*_T1_ between two T1 and the time required to drain a newly created film *τ*_drain_.

In the limit of a slow rate of T1 (*τ*_T1_ ≫ *τ*_drain_), the film is fully drained and at equilibrium at *h*_min_, while for too close T1s (*τ*_T1_ ≪ *τ*_drain_) the film has no time to drain and remains always thick, thus eventually reducing the rate of coarsening.

The average thickness is therefore set by the ratio 
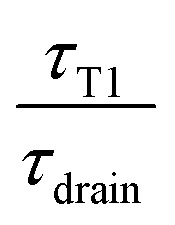
. The thickness of the film is not homogeneous, but the drainage is mostly set by the thinnest part of the film. It is the rim of the film^56^ and drains with a scaling law *t*^1/2^.

First, the thinning of the film is set by the drainage dynamics. Depending on the film thickness, viscosity, interfacial rheology and time-scale, drainage can follow different dynamics.^40,57–63^ Due to the large initial film thickness,^64^ we assume here that the drainage involves the creation of a dimple, as described by Frankel and Mysels,^56^ and by Joye *et al.*^57^ With these conditions, the drainage rate is set by the time scale3
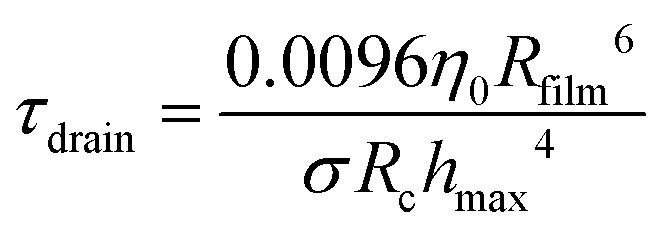
Here, *η*_0_ is the viscosity of the foaming solution assumed to be similar to water (*η*_0_ ∼ 1 mPa s); *R*_c_ is the radius of curvature of the meniscus; *R*_film_ the radius of the film; and *h*_max_ is the film thickness before drainage. This initial thickness is assumed to be proportional to the radius of curvature of the meniscus, and to follow the dynamics described in ref. 64. For a Kelvin foam of liquid fraction *ϕ*_l_ = 7%, it can be calculated that *R*_film_ ≃ 0.56*R* (such as the total area per cell divided by 12 films is equal to π*R*_film_^2^) and *R*_c_ ≃ 0.36*R*, as detailed in ref.1 and 12. W consider here only capillary drainage as it dominates over gravity effects, in the range of bubble sizes and capillary pressures. Other drainage dynamics have been described in the literature, but they describe other stages of the drainage. For instance, the Reynolds equation (which leads to different dependencies in *R*) described the drainage of an interface which is maintained flat by the van der Waals forces (disjoining pressure), which is relevant only in the last stage of drainage, while only the first stage of drainage is relevant in the present situation. Note also that, due to the complex chemical composition of the foaming solution (high concentration of surfactants and co-surfactants, together with polymers), we expect a low interfacial mobility, consistent with the drainage model we use.

Second, the time between two topological rearrangements *τ*_T1_ for a foam under shear has been shown by Gopal and Durian^16^ to be4
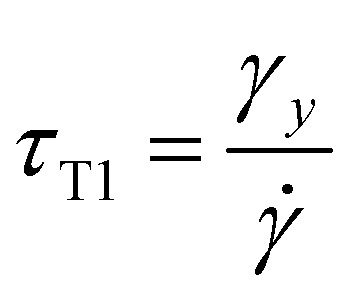
where *γ*_y_ is the yield strain of the foam, which is of the order of 0.2. Eqn (4) is valid only if the shear generates significantly more rearrangements than the spontaneous rearrangements. This condition is valid if^16^*τ*_T1_ ≪ 20 s, which is always the case under these experimental conditions. Hence, the dynamics described by *τ*_T1_ can be equivalently explored by varying the shear rate *γ̇*.

**Fig. 8:**
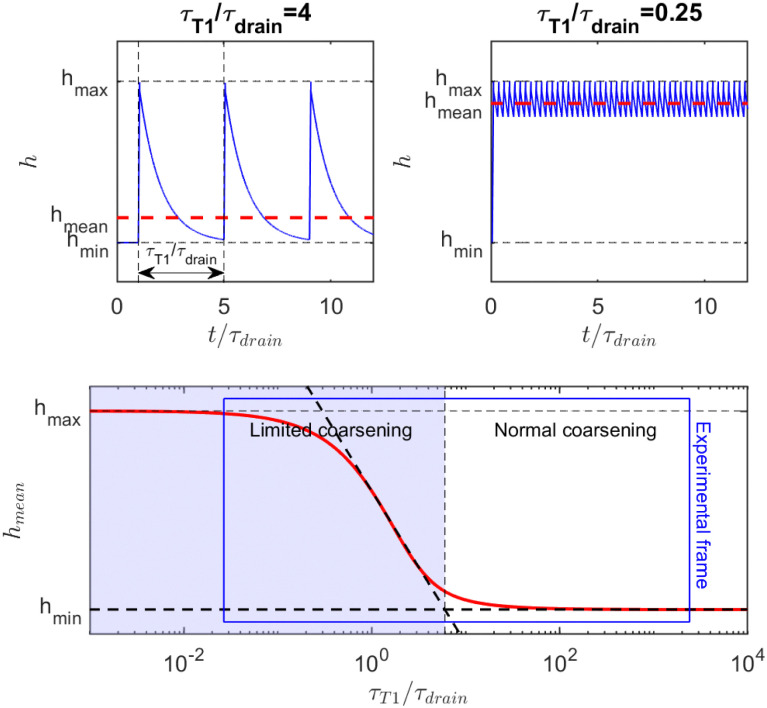
Model for the evolution of the film thickness in a foam under shear: (a) at low shear rate, the film has time to drain before being renewed by a T1; (b) at high shear rate, the film does not fully drain before a new T1 occurs. (c) Evolution of the time-average film thickness, between its two limits, as a function of the ratio of the two timescales (drainage and renewal by T1). The blue-shaded area represents the range of shear rate where the average film thickness and hence the coarsening is tuned by the shear rate. The blue frame delimits the range of parameters explored in Fig. 6.

Film-thickness evolution during a series of film creation and film drainage can be understood through a toy model shown in Fig. 8: in a first-order approximation, film drainage is assumed to follow an exponential dynamics in a time *τ*_drain_, and every *τ*_T1_, the film restarts from its initial thickness.

In the example shown in the top left plot of Fig. 8, there is enough time between T1 to drain the film (
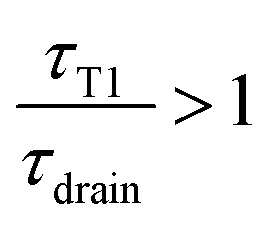
). In this limit of low frequency of T1s (low shear rate), the film remains close to the minimum value. In the top right plot of Fig. 8, T1 occurs too often (
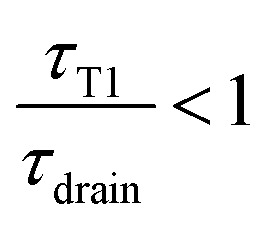
) so that the film thickness remains always close to its maximum value.

Because the coarsening rate is inversely proportional to the film thickness, we defined the average thickness as

5 *h*_mean_ = (〈*h*^−1^〉_*t*_)^−1^

where 〈·〉_*t*_ is the time average. In other words, *h*_mean_ is therefore the thickness associated with the mean 〈*D*_eff_〉.

In this exponential toy model, the inverse-averaged thickness can be calculated and is equal to6
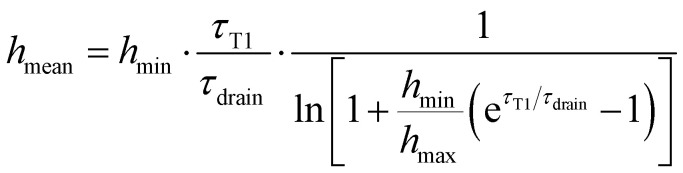
and is plotted on the bottom graph of Fig. 8: the thickness is a function of the ratio of timescales, and the transition between the minimum thickness and larger thicknesses occurs around 
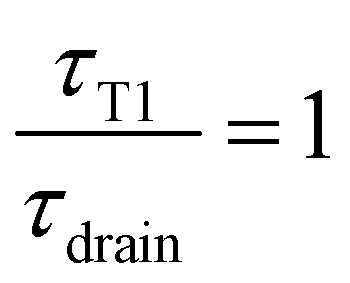
. The simplicity of this exponential toy model prevents precise quantitative comparison of the dynamics, but we apply the criteria of the equality of the drainage time scale with the rearrangement time scale to the experimental results.

As is visible in Fig. 8, the equality of the time scales (*τ*_T1_/*τ*_drain_ = 1) is located the middle of the transition range between *h*_min_ and *h*_max_, while the experimental critical shear rate is defined as the beginning of this transition, as visible in Fig. 6. The crossover of the minimum-thickness asymptote, and the variable-thickness asymptote on Fig. 8 is located at the beginning of the transition (observed experimentally) around 
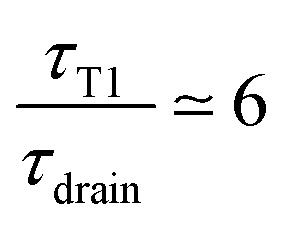
. Accordingly, we define the threshold shear rate as7
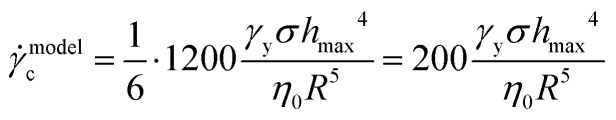
where the numerical prefactor results from the geometrical factors between the different radii, as discussed earlier.

This equation enables a quantitative estimation of the threshold shear rate *γ̇*^model^_c_: considering a film thickness after the rearrangement *h*_max_ = 0.36 mm (not a fitting parameter, based on ref. 64 rescaled by *R*_c_), eqn (7) yields an order of magnitude for *γ̇*^model^_c_ of 1 s^−1^, which matches with the experimental measurements shown in Fig. 7. It must be pointed out that *h*_max_ has a power 4 and therefore a great influence on the critical shear rate. The value of *h*_max_ = 0.36 mm comes from controlled experiment on macroscopic films of water + glycerol + sodium dodecyl sulfate.^64^ The thickness of their film converges toward 6 mm in 50 ms after the T1, and is supposed to be proportional to the meniscus radius. Taking into account their meniscus radius (280 mm) and the present one (17 mm) yields *h*_max_ ≃ 0.36 mm.

Beyond the numerical value, this model also yields dependencies on various parameters such as the bubble radius. Eqn (7) predicts a proportionality of *γ̇*^model^_c_ with *R*^−5^ in good agreement with our experimental data as presented in Fig. 7.

Taking into account the drainage of film is – to our knowledge – the only element which give such a strong dependence on *R*. Fig. 7 also shows that the investigated shear rates are significantly higher than the threshold below which the shear rate does not modify the number of rearrangements.

Thus, these agreements tend to show that the presence of thick out-of-equilibrium films is the reason for the reduced coarsening rate.

**Fig. 9:**
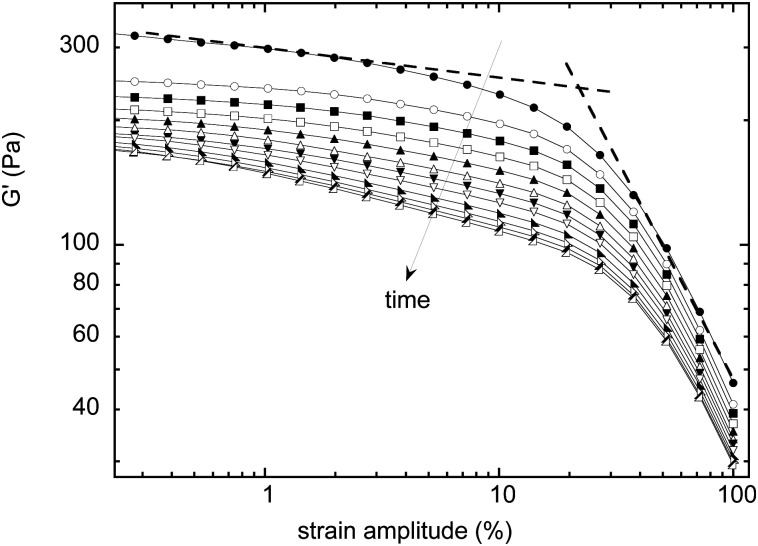
amplitude sweep experiments at different aging times *t* ∈ [200, 10 000], from which the yield strain can be extracted: it corresponds to the intersection of the two dotted lines.

Back to eqn (7), the yield strain is an important parameter in our model, as it sets the rate of T1 rearrangements. One can then wonder if it depends on the foam age, and thus on *R*. We performed experiments allowing us to infer its value, and how it evolves over time. To get *γ*_y_, amplitude-sweep tests provide us with the elastic modulus as a function of the amplitude. Then, the yield strain, *γ*_y_, is defined as the threshold amplitude above which the elastic behavior is no longer valid. Practically, we can define it as the intersection of the two dotted lines in Fig. 9, representing the two extreme regimes. It can also be defined as the first deviation from the plateau of *G*′, or from the loss of linearity of the stress curves. Whatever the criteria, it appears to be difficult to conclude how the yield strain evolves in time, thus with *R*. There might be a small evolution with *R*, which might have to be taken into account (possibly explaining the faster decrease of the threshold shear rate with the bubble size).

### 4.2 On the evolution of *G*′′

Following our interpretation of the variation of *G*′ in terms of reduced coarsening and film thickening, we can turn to the viscous modulus *G*′′, to test if the same elements are relevant for its variations.

In previous works on quiescent foams,^3^ it was shown that *G*′′ decreases with time, at least in the first two hours, in the same range of frequencies used here. Thus, for a foam at rest, *G*′′ is expected to decrease as the bubbles grow in size.

But, even a reduced coarsening, due to the applied shear, still provides an increase of bubble size, and thus *G*′′ should decrease with time, if it were mostly dependent on the bubble size. Moreover, the effects, shown in Fig. 4, of the previously applied shear on *G*′′ are found at all shear rates; there is no threshold value, as found for the evolution of *G*′. Therefore, the creation of thick out-of-equilibrium films under shear does not seem to be connected to the behavior of *G*′′. Also, shortly after the end of a steady-shear sequence, one can expect that all the films have time to drain and to get back to their equilibrium values.

One can then wonder about what is occurring during the steady-shear intervals which can then change the subsequent measurement of *G*′′, if it is not related to reduced coarsening and film thickening. Note also that the first steady shear interval has the strongest effect on the frequency dependence of *G*′′, while the impact of the other intervals is more moderate.

Even if we are not able to explain the observed features, we believe that a crucial point here is actually related to the effect of the steady shear intervals on the rate of T1 rearrangements. In a previous work on similar shaving foams, it was shown that after a transient strong shear, the duration between T1 is increased, and it then gets back to its initial value within minutes.^18^ Indeed, the applied shear relaxes all the previously stored stresses and triggers many T1, rejuvenating all the bubble arrangements and films. Moreover, *G*′′ is directly related to these T1 events and to their rate of occurrence as they are a major source of dissipation at the bubble-size scale. It is thus possible that, thanks to this new experimental protocol and as we apply the subsequent steady shear intervals, we are able to investigate other modes of dissipation, usually hidden by the constant presence of T1. Nevertheless, there still has to be some effects of the T1s, as coarsening occurs during the frequency-sweep measurements, finally triggering more and more T1. At this stage, more experiments and other experimental protocols are needed to fully determine the origins of the increase of the viscous modulus with time, and its scaling dependence with frequency.

### 4.3 On the evolution of the viscosity

Here again, we can check if the viscosity measurements, as a function of time and applied shear (Fig. 5) can be explained by the same arguments used for *G*′ and for the foam coarsening reduction. Experiments show that the viscosity decreases with time, for all shear rates. This simply appears to be a direct consequence of coarsening and of the increase of the bubble size. In that respect, one could expect that a slower coarsening (low variations of the bubble sizes) implies a smaller decrease of viscosity with time. In contrast, in Fig. 5, we find that the higher the applied shear rate (meaning low reduced coarsening), the stronger the decrease rate of the viscosity. Thus, arguments based only on bubble size variations cannot explain these measurements.

However, as shown before, the applied shear also induced thick out-of-equilibrium films between bubbles. This thickening has an impact of viscous dissipation: the thicker the films (for high applied shear rates), the lower the dissipation and the viscosity. The only way to rationalize our viscosity measurements is to add this thickening of the films under shear in the interpretation. Compared to a reference sample, the high values of shear rate induce a reduced coarsening, meaning both smaller bubble sizes (increasing *η*), but also thicker films (reducing *η*). Looking at Fig. 5 and on how the decrease rate of the viscosity depends on the shear rate, it turns out that it is this film thickening effect which dominates. We believe that this feature is another indirect proof of the existence of this film thickening under shear.

### 4.4 Behavior at high shear rates

While increasing the shear rate, the film thickness may eventually reach *h*_max_; however, other effects occur. First, another critical shear rate – identified by Gopal and Durian^16^ – has been defined, and corresponds to a crossover between localized and continuous flow. It corresponds to the shear rate at which the time between rearrangements becomes equal to the rearrangement duration itself (0.1 s according to Gopal and Durian^16^). Thus, this should occur at slightly higher rates than the threshold for film thickening.

Secondly, a high shear rate might breakup bubbles, leading to a decrease in bubble size. It has been shown that this happens when the shear stress *η*_foam_*γ̇* exceeds a critical value 
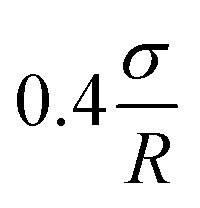
, defined by ref. 65 where *σ* is the interfacial tension of the solution (30 mN m^−1^) and *η*_foam_ is the viscosity of the foam, whose value depends on γ̇.^16^

This yields a critical shear rate for bubble breakup *γ̇*_brk_ of the order of 25 s^−1^. This shows that the shear rate that hinders coarsening, *γ̇*_c_ is well below the threshold for bubble breakup, as shown in Fig. 7.

On the other hand, the order of the magnitude of *γ̇*_brk_ suggests that the inversion and final decrease of bubble radius observed for the highest shear rates in Fig. 3 could be explained by bubble breakup.

## 5 Controlling coarsening by varying the shear rate sequence

We previously presented data obtained with a constant shear rate along one experimental run. This opens the possibility for more complex sequences of shear rates to modulate the time evolution of the bubble size. We show here two examples of how different consecutive values of shear rates can lead to different routes of coarsening.

In the first sequence called ‘high–low–high’ (HLH), we applied 8 levels of shear, separated by the frequency sweep measurements. The values (in s^−1^): 10; 0.03; 0.03; 0.03; 5; 5; 5; and 12. In the other sequence, ‘low–high–low’ (LHL), the values of shear rates are: (in s^−1^): 0.03; 5; 5; 15;15; 0.03; 0.03; and 0.03.

**Fig. 10:**
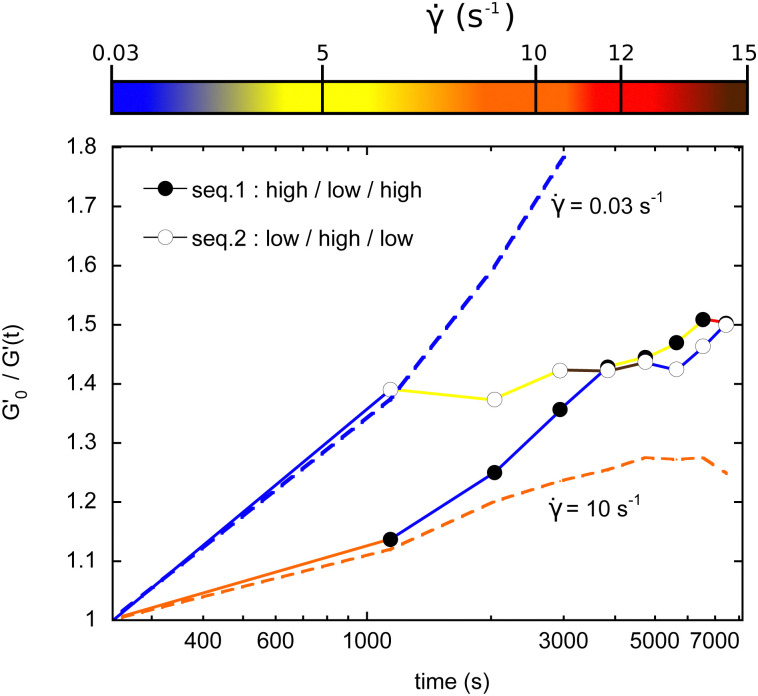
Different routes for adjusting the coarsening rate between the two extreme limits.

In Fig. 10, one can unambiguously ascribe each consecutive portions of high and low coarsening to the shear rate range. For the HLH sequence, coarsening is first hindered, then bubbles grow faster, and finally this growth is again reduced. The opposite behavior is consistently found for the LHL sequence. This clearly demonstrates that non monotonous bubble growth rate can be obtained by modulating the applied shear. We also plotted the two extreme limits corresponding to the measured behaviors should the shear rate have been kept constant at the two initial values of the sequences, 0.03 and 10 s^−1^.

As a last comment on these experiments, note also that the same bubble sizes can be finally reached after two hours, but *via* two different routes.

## 6 Conclusions

Thanks to a new rheological protocol and based on the measurement of the elastic modulus, we have monitored how an aqueous foam coarsens under shear and have evidenced that an applied shear actually slows down coarsening. This effect occurs above a shear rate threshold, strongly dependent on the bubble size. We propose here an interpretation relying on a shear-dependent thickness of the films separating the bubbles. The threshold results from the competition between the dynamics of bubble rearrangements and of film drainage. For shear rates above the threshold, the time to rearrange becomes too high compared to the time to drain a newly created film, and an out-of-equilibrium film thickness can then be dynamically sustained. The strong dependence with the bubble size arises from the dynamics of film thinning. We also show that this film thickening has an impact on how foam viscosity depends on time and shear rate. Regarding the understanding of foam flows at the local scale of films and bubbles, these results provide us with a new element, and help us to complement the sequence of flow phenomena occurring as the shear rate is varied. Indeed, we still have to elucidate the possible links between this film thickening and the subsequent foam melting observed by Gopal and Durian.^16^ On these issues, these results also show that shear actually induces some redistribution of liquid within the liquid skeleton, which can then have macroscopic signatures. In parallel, there are still many pending issues, for instance on the dependence of these effects on the interfacial rheology (which can change the film thinning dynamics), and on the foam liquid fraction. Also, these results open many questions on viscous dissipation and on the time evolution *G*′′, which is different from the one measured when no shear is applied. More generally, these results and the new protocol proposed could be relevant and useful for the rheology of other soft materials like concentrated emulsions or microgels. Finally, on a practical point of view, we also demonstrated that non-trivial and non-monotonous bubble growth rate could be obtained by controlling the sequence of applied shears.

## Author contributions

ASJ designed the experimental protocol and performed the experiments. CT and ASJ developed the theoretical model, analyzed the experimental results and wrote the manuscript.

## Conflicts of interest

There are no conflicts to declare.

## Supplementary Material

SM-019-D2SM01618D-s001
