# Supplementary material for: Foam coarsening under a steady shear: interplay between bubble rearrangement and film thinning dynamics
Source: Soft Matter. 2023 Feb 17;19(11):2090–8. doi: 10.1039/d2sm01618d (PMC10015626; doi:10.1039/d2sm01618d)
Supplement: SM-019-D2SM01618D-s001 [file SM-019-D2SM01618D-s001.pdf]

Electronic Supplementary Material (ESI) for Soft Matter.
This journal is © The Royal Society of Chemistry 2023

Supplementary material for ”Foam coarsening under steady shear : interplay between bubble rearrangement and film thinning dynamics”

Arnaud Saint-Jalmes & Corentin Trégouët

I. bubble size evolution by microscopy

Our interpretation of the rheological measurements presented in the article is that an imposed steady shear can reduce the coarsening rate, so that the bubble size actually depends on the shear rate.

To corroborate this interpretation, we propose to add an independent estimation of the bubble size. Note that a direct and unambiguous measurement of the bubble size within such foams is not a simple task. Here, we have chosen to use the bubble raft technique. It consists in collecting some bubbles from the interior of the foam and depositing them on a thin solution layer, so that the bubble creates a raft of one bubble thickness, with all the bubbles well separated so that their radii can be measured, as shown in Fig. S1.

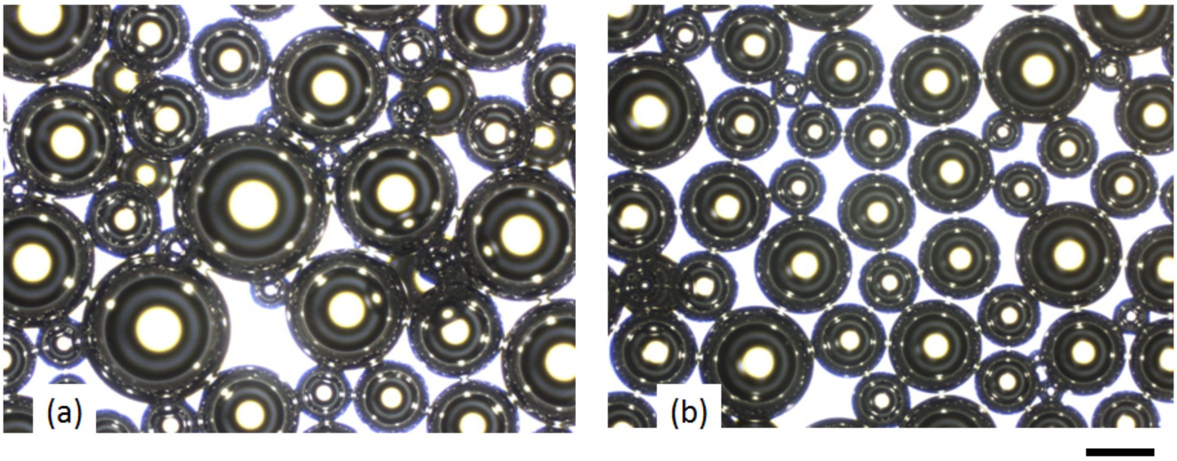

Fig. S1: Bubble rafts after shearing at low shear rate (0.05 s^{-1}) on the left or high shear rate (5 s^{-1}) on the right.

Nevertheless, there are drawbacks to this approach need to open the plate-plate confining geometry, all the required actions (collection with a spoon, deposition on solution, etc...) are intrusive and may change the bubble size; statistics is low (small number of bubbles compared to the number involved in the elasticity measurement). On that last point, collection of bubbles and imaging are performed at least ten times, creating various different bubble rafts.

Two sets of images were then analyzed: one taken after two hours of continuous shear at a rate of 0,05 s^{-1}, and the other one after two hours at a rate of 5 s^{-1}. Typical images are given in Fig. S1, and the averaged bubble radius is then extracted and given in Fig. S2 which compare the volume-averaged radius, for low and high shear rate. Standard deviation σ_{std} is shown by the thin error bars: however large the standard deviation, the large number of measurements (N) enables a precise estimate of the mean radius. The error estimate on the mean radius is $\sigma_{std}/\sqrt{N}$ and is shown by the thick error bars. Moreover, Student T-test yield a p-value of 0.0016<0.05, confirming the relevance of the difference between the mean radii. It results that the optical direct measurement corroborates the elasticity measurements: shearing the foams delays the coarsening.

This analysis also shows that the standard deviation is significantly smaller in the case of high shear. This could be the signature of the dependency of the shear-rate threshold on the bubble radius: if bubbles can

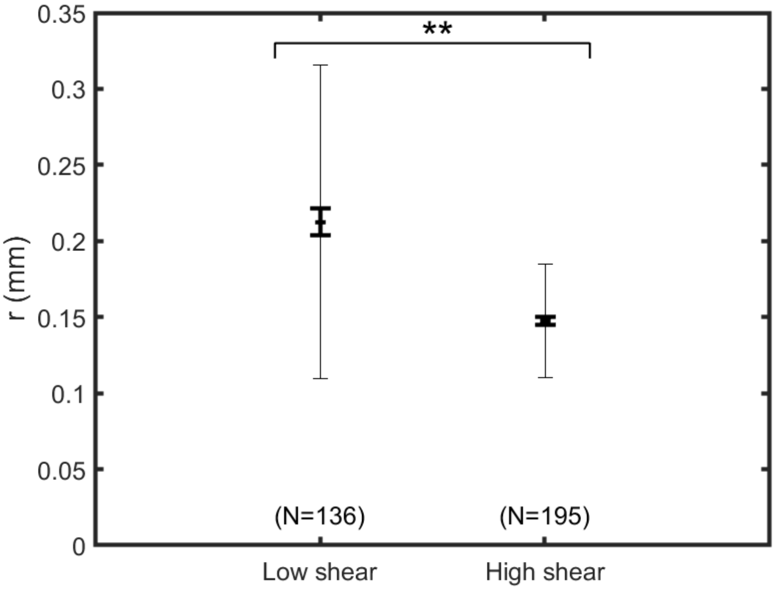

Fig. S2: Bubble size with low (0.05 s^{-1}) or high (5 s^{-1}) shear rate. The average is calculated weighed by the bubble volume. Thin error bars represent the standard deviation of the size distribution, and the thick bars represent the error on the calculated mean value (standard deviation of the sample divided by the number of bubbles). Student's T test yield a p-value of 0.0016.

coarsen at a normal rate and grow until a threshold radius (corresponding to the applied shear rate) where the coarsening slows down, most of the bubbles should reach a size close to the threshold radius, decreasing the polydispersity.

II. Thickness-variation estimation

The relative thickness extracted from the coarsening early kinetics is plotted in Figure S3: it shows a thickness increase by a factor 3 due to the applied shear rate.

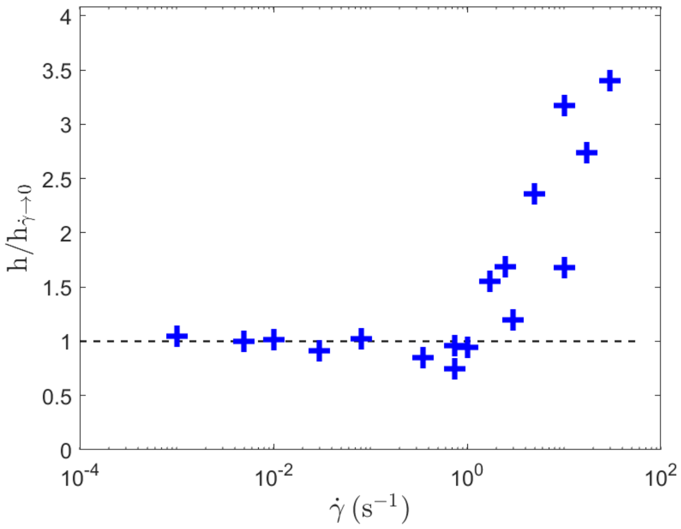

Fig. S3: Relative thickness h extracted from the initial growth rate of $(G'_0/G'(t))^2$ *with the data of Figure 3. The thickness is normalized by the observed plateau at vanishing shear rate.*
